# Exposure scenarios for human health risk assessment of nano- and microplastic particles

**DOI:** 10.1186/s43591-025-00134-9

**Published:** 2025-07-14

**Authors:** Taylor Lane, Ira Wardani, Albert A. Koelmans

**Affiliations:** Aquatic Ecology and Water Quality Management Group, Department of Environmental Sciences, Wageningen University, Wageningen, 6700 AA The Netherlands

**Keywords:** Microplastic, Exposure pathways, Data alignment, Demographic assessment

## Abstract

Evidence of nano- and microplastic particles being present in the human body has increased in recent years, yet there is no acceptable methodology to perform a human health risk assessment for these particles because of limitations in the exposure and hazard assessments. Exposure assessment can be improved by establishing comprehensive and justifiable exposure scenarios for a defined exposure demographic, thoroughly describing the relevant exposure pathways, and performing multidimensional data alignment, thereby facilitating probabilistic estimates of nano- and microplastic particle exposure. General considerations of exposure scenarios are outlined, along with specifics details on the complexity and prioritization for nine demographic groups: adults; women; the elderly; individuals with disease; individuals employed in high-hazard occupations; and children demographics, including early infants, toddlers, school children, and teenagers. Recommendations to advance exposure assessments and scenarios are also provided which suggest: i) the use of well-defined exposure scenarios for demographics that are prioritized according to their level of complexity and concern; ii) a thorough description of relevant activity factors (physiological parameters, behavioural traits) and exposure factors (duration, frequency, media characterization) for the chosen demographic; iii) thorough descriptions of exposure via ingestion and inhalation, and in the case of early infants, including exposure via maternal transfer; iv) multidimensional data alignment and probabilistic methods to enable credible comparisons of exposure data across studies and inform physiologically based toxicokinetic models to estimate internal exposure.

**Supplementary Information:**

The online version contains supplementary material available at 10.1186/s43591-025-00134-9.

## Introduction

Society faces numerous unresolved questions and challenges related to the quality of our living environment and its connection to human health and well-being. Among these challenges, plastic pollution has emerged as a crisis, necessitating the development of appropriate regulatory and healthcare policies to safeguard human health [56, 106]. Once released into environment, plastics degrade through a combination of physical, chemical, and biological processes, generating nano- and microplastic particles (NMPs). For this article, nanoplastics are defined as plastic particles ranging from 0.01 to < 1 µm (µm) in size, and microplastics as plastic particles ranging from 1 to ≤ 5000 µm in size. The inevitability of human exposure to NMPs has sparked mounting concerns about potential health effects among the public, scientists, health professionals, media, and policymakers [1, 24, 93, 121, 123].

After exposure, NMPs can be absorbed into the bloodstream in a size-dependent fashion and translocated throughout the body, where accumulation in various organ systems may impair function [3]. A summary of *in vitro *effect studies suggests that intestinal cells undergo cytotoxicity, as well as impacts to barrier integrity and inflammatory responses, after exposure to NMPs approximately 6µm or smaller [13]. In lung cells, immune responses and cytotoxic effects have been observed in studies after exposure to NMPs approximately 10µm or smaller [110]. At the same time, NMPs are being detected in samples derived from various human organ systems and bodily fluids, demonstrating widespread body burden [60, 127]. Therefore, a critical problem to solve is whether increasing exposure to NMPs can be linked to short-term or long-term adverse health effects in humans [4, 21, 55]. 

To solve this problem, human health risk assessment for NMPs is needed [48, 113]. A fundamental component of the human health risk assessment process is the exposure assessment, which aims to identify the exposed population or demographic(s), determine how exposure occurs, and to quantify the extent of exposure [105]. In the case of humans and exposure to NMPs, everyone is exposed through the air, water, and food – but how does the magnitude of exposure vary amongst human demographics of different ages, lifestyles, and geographic locations? Assuming a “one-size-fits-all” approach to describe human exposure to NMPs is imprudent because vulnerable demographics might be overlooked, although the use of safety factors in the assessment might provide a level of protection for these demographics.

**Fig. 1:**
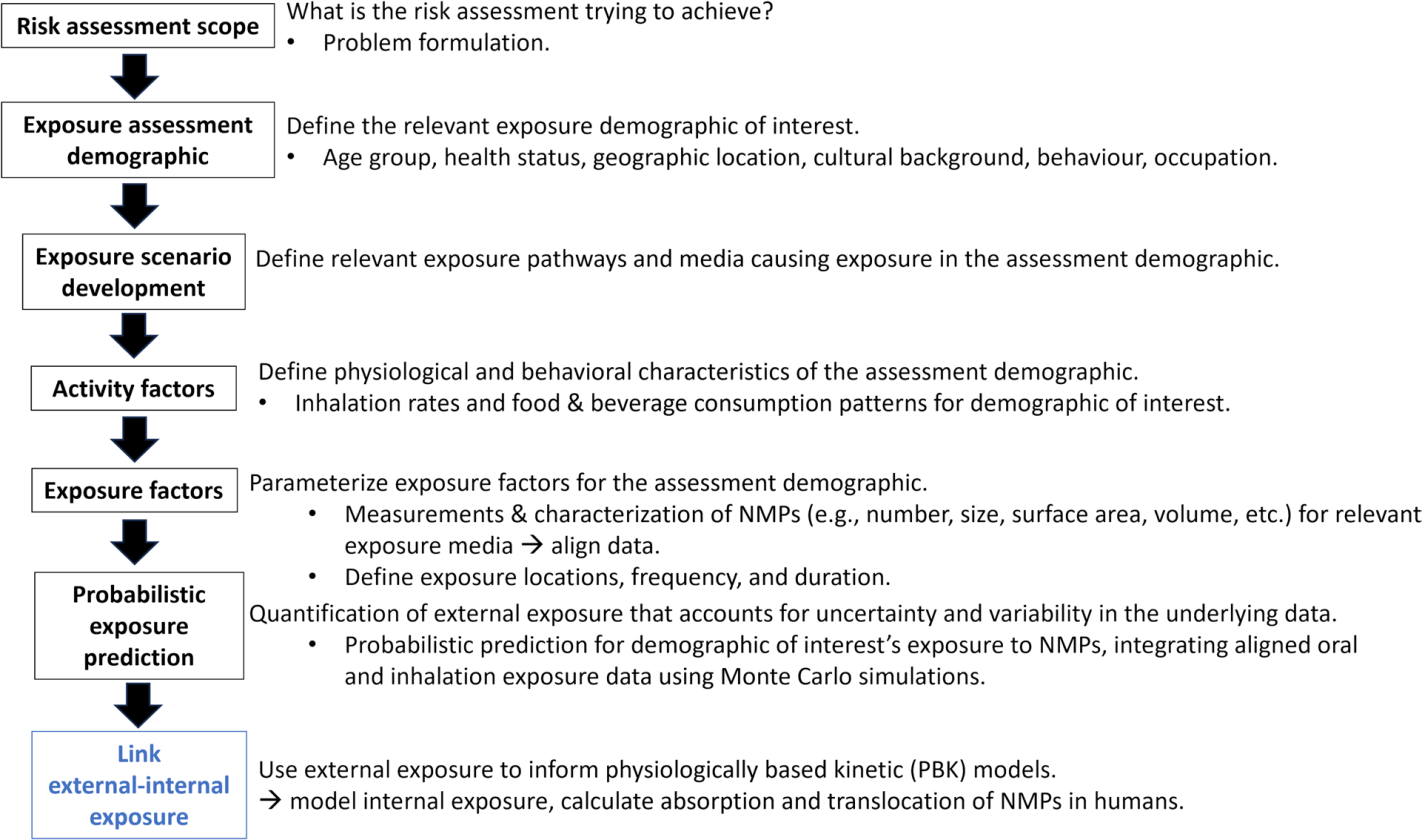
Pathway of exposure assessment and scenario development for NMPs. The exposure assessment is defined and used to describe the demographic of interest. Specific exposure scenarios are then developed, and data is collected to parameterize the necessary activity and exposure factors for the exposure demographic. All the data, assumptions, uncertainty, and variability in the activity and exposure factors are integrated into a probabilistic model to estimate the likelihood and range of potential exposures. Results of the external exposure predictions are used to inform physiologically based toxicokinetic models which estimate internal exposure (i.e., body burden in different organs or fluid compartments)

Rather than using a “one-size-fits-all” approach, exposure scenarios can be developed to more rigorously estimate human exposure for a demographic of concern. Exposure scenarios are termed as “a combination of facts, assumptions, and interferences that define a discrete situation where potential exposures may occur” [119]. The exposure scenario is defined by relevant activity and exposure factors, reflecting the facts, assumptions, and interferences specific to the demographic under assessment. Clearly defined exposure scenarios for NMPs link the overarching goals of the risk or exposure assessment to the relevant demographic-specific exposure information, primarily the activity and exposure factors, thereby facilitating probabilistic exposure assessments (Fig. 1).

Exposure scenarios provide a framework to calculate external exposure across different demographics or subpopulations of people. In addition, the fraction of particles that are taken up or absorbed by the body must be described. Physiologically based toxicokinetic models can be used to predict an internal exposure or body burden. External exposure is defined as what NMPs humans are exposed to orally, from the ingestion of food, beverages, the incidental ingestion of dust, soil, and airborne particles trapped in mucus, as well as exposure to NMPs from the inhalation of indoor and outdoor air. Internal exposure is defined as the fraction of external exposure absorbed into the human body and distributed therein.

Activity factors incorporate aspects of human psychology, physiology, and health status of the scenario demographic which informs exposure factors used in the probabilistic exposure calculations [38]. Activity factors will also include food consumption rates and air inhalation rates, which vary according to age, physiology, geographical location, behaviour, and other potentially relevant factors. Exposure factors provide information on exposure pathways, and the composition and variability of NMPs (e.g. size range, number, shapes, surface area, concentration, etc.) within specific exposure media, as well as exposure frequency, duration, and location. Certain particle characteristics, such as surface area for particles reaching the lungs, might be more toxicologically relevant than other characteristics, like particle size, volume, or concentration [94]. Exposure to toxicologically relevant characteristics of NMPs can be described probabilistically by constructing distributions of specific particle characteristics across various exposure media [48].

Scenarios for human health risk assessment of NMPs should capture the diverse, widespread, and chronic nature of exposure, which primarily occurs through inhalation of air and ingestion of food and beverages, leading to inhalation and oral exposure. A third recognized exposure route is dermal, most associated with cosmetics or personal care products; however, evidence suggests that only particles smaller than 45 nm (nanometer) can penetrate the skin [34]. Although the dermal exposure route warrants future exploration, oral and inhalation exposure are currently considered more prevalent, and for early infants (age 0–1 year old), maternal transfer is also important; thus, our focus is directed towards these exposure routes. Despite recognition of the importance of these exposure routes, most human exposure assessments for NMPs conducted to date have used data that inadequately characterizes exposure and have employed incomplete exposure scenarios.

The aim of this perspective paper is to cultivate constructive discussions around the assessment of human exposure to NMPs. The paper begins with a summary of general considerations for exposure scenarios and how exposure scenarios have been used thus far to estimate human exposure to NMPs, highlighting the strengths and weaknesses of various study approaches. Then, exposure scenarios are elaborated for the most prevailing demographics including adults (ages 18–65 years), women, the elderly (ages 65 and over), individuals with underlying diseases, individuals who work in potentially hazardous occupations, and sub-groups of children, such as early infants (age 0–1 year), toddlers (ages 1–4 years), school children (ages 4–13 years) and teenagers (ages 13–18 years). For each demographic’s exposure scenario, a description of the relevant activity factors, linkages between activity factors and exposure factors, and the impact the selected activity and exposure factors may have on the exposure prediction are discussed. Furthermore, a ranking of complexity and prioritization for each of the exposure scenarios is given. And we provide suggestions to improve estimates of human exposure to NMPs, such as proper alignment and rescaling of exposure data from different studies, the integration of toxicologically relevant metrics for characteristics of NMPs, a thorough incorporation of exposure pathways, and the application of probabilistic approaches to link external-internal exposure using physiologically based toxicokinetic models. Lastly, we reflect on current research needs with recommendations for future work.

### General considerations for developing exposure scenarios

There are general considerations for exposure scenarios for NMPs that provide a foundation of factors applicable to all demographic groups. Exposure pathways should be defined for oral exposure from ingestion of food and beverages, and for inhalation exposure from different sources of indoor and outdoor air. The frequency of exposure must also be considered across all exposure scenarios, such that acute and chronic exposure situations can be separated, with the frequency of exposure being defined on an appropriate temporal scale. Additionally, a decision should be made regarding the level of caution applied in the exposure scenario, specifically determining whether predictions should reflect average, realistic, or worst-case conditions. For instance, a worst-case prediction using 95th percentile exposure data could provide a more precautionary assessment compared to using 50th percentile exposure data. Demographic and physiological factors also play a role in all scenarios with age, sex, body weight, inhalation rates, and physiological development all influencing exposure. Metrics for activity factors are also available, such as Beyer et al. [11] and Chaput et al. [18], which define the time spent outdoors and sleeping, respectively, for children, adults, and other age sub-groups.

Additionally, a cross-cutting aspect present in all scenarios are differences between exposure demographics with regards to their behavioural, regional, and cultural activity factors that impact their lifestyle patterns and thus exposure to NMPs. For example, exposure could be substantially affected for those following a vegetarian or vegan diet [28, 70, 109], compared to those who practice certain cultural or religious diets such as halal, kosher, or Ramadan dietary regimens [22, 43, 92]. Exposure could also be altered based on the socioeconomic status and geographical location of a given demographic [136]. Socioeconomic factors and geographical location can affect the type and quality of food items being consumed [33, 41], as well as the amount of time spent in locations that are prone to elevated or decreased concentrations of NMPs in the air. Scenarios should consider life stage specific factors in this regard (e.g., adult versus children). As well, exposure can be interlinked in various ways, and multi-route combinations and interactions should be accounted for when possible. This concept is discussed later in the paper (see section “Thorough characterization of exposure pathways”).

All scenarios must consider the uncertainty in the underlying data, as well as the quality control and quality assurance (QA/QC) of the data informing their predictions. Factors such as different analytical detection limits, study design, the use of controls, and sample amounts analysed for NMPs can impact the reliability and variability of exposure measurements across different studies [46, 124]. Nevertheless, once ample data are available that characterize NMPs in the environmental media responsible for exposure, an understanding of the distribution of particle characteristics can be gained. Methods to estimate exposure based on particle mass or other particle metrics, such as particle number, volume, or specific surface area, are applicable to all scenarios. Furthermore, conversions of one metric to another, such as from mass to number, or vice-versa, and the calculation of other dose metrics (e.g., surface area) is applicable to all scenarios. Exposure scenarios can always integrate a multidimensional alignment (see the “ Multidimensional exposure data alignment” section for details) of exposure data in their predictions. Another factor all exposure scenarios might consider are implications of microbial or bio-corona associations with NMPs and how these could impact both internal and external exposure. As well, there are also risks of non-plastic based nanoparticles and microparticles (e.g., black carbon particles), as discussed in Koelmans et al. [48].

Scenarios should therefore aim to provide estimations of combined exposure that integrate all relevant pathways for risk assessment. In this way, external exposure can be properly characterized and aligned with internal exposure using physiologically based toxicokinetic models, providing a bridge to hazard and insights regarding potential health risks. Then, risk assessment approaches, such as broadly applicable one’s like the International Life Science Institute’s, Health and Environmental Science Institute’s Risk 21 concept [122], or frameworks specific to NMPs such as Wardani et al. [117] can be used.

The above considerations, while applicable to all scenarios, may carry different weight in certain scenarios. For instance, exposure to plastic particles via infant formula or breastmilk is applicable to early infants (age 0–1 year old) and not to adults. Or hazardous occupational exposure to airborne NMPs is more applicable to adults working in these types of jobs and not to early infants or school children. As well, certain demographics might require focus on specific pathways, such as maternal transfer in the case of mother and early infant. In Table 1, a view on the similarities and differences in exposure media across different exposure demographics is provided.

**Table 1:** Relevance of exposure routes and media for different human exposure demographics

Exposure Routes & Media	Exposure demographics								
	Adult (18–65 years)	Women	Elderly (65 + years)	Individuals with underlying disease	Individuals working in hazard occupation	Early infant (0–1 year)	Toddler (1–4 years)	School child (4–13 years)	Teenager (13–18 years)
Inhalation of NMPs from residential setting	●	●	●	●	●	●	●	●	●
Inhalation of NMPs from hazardous job setting^a^	-	-	-	?	●	-	-	-	-
Inhalation of NMPs from school setting	?	?	-	?	-	-	-	●	●
Inhalation of NMPs from different outdoor air sources (urban or rural)	●	●	●	●	●	●	●	●	●
Inhalation of NMPs from other indoor settings	●	●	●	●	●	●	●	●	●
Ingestion of NMPs from dairy products	●	●	●	●	●	?	●	●	●
Ingestion of NMPs from cereals & grains	●	●	●	●	●	-	●	●	●
Ingestion of NMPs from vegetables	●	●	●	●	●	-	●	●	●
Ingestion of NMPs from fruits, nuts & seeds	●	●	●	●	●	-	●	●	●
Ingestion of NMPs from meat	●	●	●	●	●	-	●	●	●
Ingestion of NMPs from seafood/fish	●	●	●	●	●	-	●	●	●
Ingestion of NMPs from eggs	●	●	●	●	●	-	●	●	●
Ingestion of NMPs from fats & oils	●	●	●	●	●	-	●	●	●
Ingestion of NMPs from sugar	●	●	●	●	●	-	●	●	●
Ingestion of NMPs from salt	●	●	●	●	●	-	●	●	●
Ingestion of NMPs depositing to food or beverage	●	●	●	●	●	-	●	●	●
Ingestion of NMPs from tap water or bottled water	●	●	●	●	●	●	●	●	●
Ingestion of NMPs from coffee & teas	●	●	●	●	●	-	●	●	●
Ingestion of NMPs from fruit & vegetable juice	●	●	●	●	●	-	●	●	●
Ingestion of NMPs from breastmilk	-	-	-	-	-	●	-	-	-
Ingestion of NMPs from infant formula	-	-	-	-	-	●	-	-	-
Ingestion of NMPs from alcoholic beer & wine	●	●	●	●	●	-	-	-	?
Ingestion of NMPs from non-alcoholic beer & wine	●	●	●	●	●	-	-	-	?
Ingestion of NMPs from particles trapped in mucociliary clearance	●	●	●	●	●	●	●	●	●

● Exposure pathway and media are relevant to the exposure demographic

? Exposure pathway and media potentially relevant to the exposure demographic

- Exposure pathway and media are not relevant to the exposure demographic

^a ^Hazardous job defined as an occupation where elevated amounts of airborne NMPs are generated due to workplace production and/or processing of materials (e.g., textiles, plastic product manufacturing or processing)

### Additional considerations applicable to exposure scenarios for children

A famous quote from the Swiss philosopher, Jean-Jacques Rousseau, eloquently states that “Children are not small adults”. Children are a unique demographic of the human population because their behaviour, physiological development, and dietary consumption patterns differ not only from adults, but also amongst the various periods of a child’s development. Children are generally defined as individuals aged 0–18 years old. For this article, smaller, age-based categories are used, which are early infants (0–1 year), toddlers (1–4 years), school children (4–13 years), and teenagers (13–18 years).

Compared to adults, children generally have relatively greater exposure to NMPs. For instance, children have higher metabolic rates to support their growth and development, which can be observed through measures of total daily energy expenditure, and greater caloric consumption per body mass [87]. Consumption of more food by children can lead to a relatively higher exposure on a per body mass level. Respiratory differences also exist, such as fewer and smaller alveoli, until the age of 8, which limits gas exchange and the amount of oxygen entering the blood stream, thus requiring increased rates of respiration [132]. The respiratory difference is clearly observed in the resting oxygen consumption of a newborn baby being 6 mL/kg/min which is two-fold that of an adult at rest which consumes oxygen at 3 mL/kg/min [132]. Inhalation rate selection can therefore have considerable implications when calculating exposure to airborne particles [30].

There are also age-specific dynamics to exposure scenarios for children. During their toddler years, children are likely to interact with the environment more intimately, as they crawl close to the ground, or practice mouthing behaviour, which is a hand-to-mouth action that can lead to the incidental ingestion of dust and dirt, and other contaminants that are present on their hands and objects [98]. Defining these activity factors is worthwhile because evidence indicates the presence of NMPs in household dust [77, 134], as well as the sand and soil in children’s playgrounds [54]. The amount of time children spend in these locations, as well as their behaviour, will impact their exposure [25, 73].

The circumstances associated with children and exposure to NMPs have been reviewed extensively by Sripada et al. [98], however the authors did not exclusively discuss or define exposure scenarios in children. Rather, they called upon researchers to explore children’s exposure to NMPs “comprehensively and quantitatively”, which can be resolved through exposure scenarios. Developing exposure scenarios requires the identification of age-specific activity and exposure factors. In a report by van Engelend and Prud’homme de Lodder [107], about assessing exposure in children, two key screening questions were presented as important considerations when evaluating child-specific exposure:

1. Is the source of exposure accessible to children?
2. Do child-specific activities indicate a possible route of exposure which might not be relevant for adults?

Building on these screening questions and the recommendations of Armstrong et al. [6], it is essential to examine exposure to NMPs in children, with respect to their different stages of development, in the following categories:

- Regularly consumed food and drink (ingestion).
- Residential, school, and outdoor air (inhalation).
- Soil and dust in areas consistently frequented (incidental inhalation and possibly ingestion).
- Maternal transfer (mother to early infant only).

Across all children developmental stages, the physiological and behavioural circumstances imply potential for acute and/or chronic adverse health outcomes from exposure to NMPs. Risk assessments must explicitly account for these vulnerabilities. Effects might manifest early or later in life, possibly due to developmental disruptions or the accumulation of NMPs over time. It is important that these areas of research are explored in the future to protect children from exposure to NMPs.

### Current state of exposure assessment and scenario approaches

Reliably quantifying human exposure to NMPs remains a formidable task, even with a substantial understanding of the relevant exposure pathways, routes, and media. Some of the earliest studies (e.g., [27, 96]) calculated an average exposure based on controversial assumptions, as detailed by Pletz [86]. Other studies predicted human exposure to NMPs for a limited fraction of possible exposure media [26, 70, 133, 137]. Recent studies included factors related to human behaviour [62] or differences in geographical locations [136]. However, none of the above studies provided their exposure calculations using aligned and re-scaled data, as done in Mohamed Nor et al., [71] and Chen et al., [19]. In Table S1, a summary of studies predicting human exposure to NMPs is provided. Please note, this list of studies is not meant to be exhaustive, as a formal literature search was not conducted. These studies are meant to serve as examples of different approaches authors have used to estimate human exposure to NMPs.

In the study by Cox et al. [27], adults (≥ 19 years old) and children (1–18 years old) were grouped as male or female, and exposure to NMPs via oral and inhalation routes was evaluated. Inhalation exposure was simply air, with no distinction between indoor or outdoor air. Cox et al., [27] also recognized that the data used to describe the composition of NMPs in air was limited to two studies containing highly variable data. The ingestion exposure included only seafood, sugar, honey, salt, and beverages like alcohol and water. In the end, Cox et al. [27] study predicted an annual exposure per person to approximately 74,000—114,00 particles from ingestion and inhalation, regardless of age or gender. The authors stated their values as being an underestimation of exposure, partly due to the omission of common dietary items in the ingestion exposure calculations. The Cox et al. [27] exposure calculations are challenging to interpret simply based on particle numbers because the combination of data used was not aligned, meaning differences in the measured particle size ranges were not considered.

The study by Senathirajah et al. [96] intended to provide a reliable prediction of exposure, and reported humans globally might be consuming approximately 0.1—5.5 g of plastic per week, or approximately 11,845—193,200 particles per year. However, this study included several deficiencies, such as the misinterpretation of units of measurement, comparisons of data (e.g., particle counts) that are not logical to compare [86], assuming all particles are spherical in shape, including a limited number of dietary items, and the omission of inhalation exposure. It was concluded by later studies [71, 86] that oral exposure to NMPs in humans was considerably overestimated in Senanthirajah et al. [96]. In fact, the exposure estimates from Senathirajah et al. [96] were above the 99th percentile of the Mohamed Nor et al. [71] distribution.

Another example is Conti et al. [26], who calculated oral exposure to NMPs in adults from ingestion of only fruits and vegetables, with no consideration of any other dietary items, nor inhalation exposure. This study first measured the number of NMPs in fruit and vegetable items they collected and used this to inform their exposure calculations. Conti et al. [26] measured an average of 132,740 particles per gram of fruit or vegetable and provided an estimated daily exposure of approximately 460,000 in adults and 1,400,000 particles in children, per kilogram of bodyweight per day. These values are much higher than estimates of exposure in other studies when expressed on an annual basis (Table S1). Domenech et al., [29] also made a prediction of average exposure to NMPs in humans through oral and inhalation, including the values used in Conti et al. [26] in their prediction, as well as data on seafood, bottled water, salt, alcohol, and air, with no distinction between indoor or outdoor air, nor with any data alignment principles applied, and simply grouping all humans into a single demographic group. The exposure prediction from Domenech et al. [29] was upwards of 29,300,000,000 particles per year, largely driven by the oral exposure route, as inhalation exposure contributed 2160 particles per year.

Two papers by Bai et al. [7, 8] provide another set of human oral exposure estimations, with no regard for inhalation exposure. Their first paper looked at a fraction of normal dietary items applicable to the general population, and in the second paper, they looked at exposure due to Chinese takeaway (takeout) food consumption. Their general population exposure was based on beverage data, with mean concentrations of NMPs for beer, bottled/mineral water, milk, wine and other beverages being used, as well the food items honey, poultry, and seafood. No stratification across ages was made and their annual exposure estimate was 142,000–154,000 NMPs per person. In their other study, Bai et al. [8] decided to stratify demographics across age groups – from toddlers to children and teenagers, adults, and the elderly. Their oral exposure only considered ingestion of the takeout food at lunch time, and the food items consisted of rice, noodles, bean products, meat, vegetables, and beverages (bubble tea and coffee), resulting in an average annual oral exposure between 8,840–33,176 NMPs per person. The predictions of Bai et al. [7, 8] are multiple orders of magnitude lower than other papers, mainly because they considered just a small portion of what humans consume. Like other papers, no data alignment was performed. Notably, Bai et al. [8] also predicted toddler exposure to bubble tea, coffee, and Chinese takeout food, and consumption of these food items is questionable for this age demographic.

In an exposure scenario defined by Zuri et al. [137], a daily meal-plan was created based on consuming a cup of milk for breakfast, fish and white wine for lunch; and for dinner, shrimps, blue mussels, and a small beer. The Zuri et al. [137] meal-plan was used to estimate the daily ingestion of NMPs for adult humans using the average concentrations in the food or beverages, respectively. This scenario is questionable because, the meal-plan used is not realistic, and therefore the relevance of this exposure estimate is not evident. At the same time, Zuri et al. [137] also evaluated inhalation exposure in adults, children, and early infants, using exposure factor assumptions for the respiratory rates, tidal volumes of the lung, and time spent indoors and outdoors for each age demographic. Their approach for inhalation exposure makes more sense than their oral exposure estimation based on their meal-plan, although for both routes, the authors did not align the data used in the predictions.

In a different study, a protein consumption survey of the United States of America population was used to estimate exposure to NMPs from different protein sources and products [70]. In this paper, the authors collected, processed, and analysed the protein products for NMPs and used this information along side their consumption survey data to make their exposure calculations. The products examined for NMPs ranged from unprocessed meats like fish, chicken, beef, and pork, to processed products like chicken nugget, plant-based meats, and tofu [70]. The focus of Milne et al. [70] was solely on processed and unprocessed meat items consumed by humans, and the author’s study design made great efforts regarding the QA/QC. Milne et al. [70] concluded that United States of America adults are exposed to more than 11,000 NMPs per year from the consumption of protein. Like other studies, this study omitted exposure from other food items, as well as from inhalation. The inclusion of data for other routinely consumed dietary components, such as dairy products, grains, fruits, and vegetables, is crucial for a comprehensive evaluation of oral exposure.

Liu et al. [62] provided a valuable study about how behavioural and physiological activity factors could impact exposure to NMPs at different ages. The Liu et al. [62] prediction concentrated on exposure from air inhalation and the ingestion of surface dust and soil, without any focus on dietary exposure. Although this paper provided a thorough description of indoor and outdoor air exposure for different countries, age groups, and behavioural scenarios, the underlying data was not aligned. A main finding from this work was that time in residential settings was a major factor linked to inhalation exposure across all age groups, from ages 1–4 to > 64 years old.

An evaluation of adult human exposure to NMPs was carried out by Zhao and You [136]. In their study, thorough ingestion and inhalation exposure pathways for NMPs were composed using recent literature data to inform an estimate of human exposure in 109 different countries, respectively [136]. Nevertheless, several assumptions were made that should be taken into account when analysing this study, such as the assumption that all NMPs causing exposure are spherical in shape, using average values of exposure data (e.g., average particle size) rather than aligned data, and assuming that inhalation rates, as well as accumulation and excretion kinetics, are static across all people globally. The authors also did not consider how age, dietary habits, and the physical characteristics of NMPs may impact exposure. Notably, the release of NMPs from plastic packaging materials to food was included in the exposure estimate of Zhao and You [136].

The study of Mohamed Nor et al., [71] was the first in which the authors implemented data alignment and made probabilistic estimations of exposure to NMPs. Mohamed Nor et al. [71] used the best available data at that time to generate analogous particle size distributions across the dietary items and airborne media considered in their exposure prediction. Mohamed Nor et al. [71] calculated a median exposure estimate of approximately 202,000 NMPs/year and 323,000 NMPs/year in adults and children, respectively, while only accounting for 20% of the diet by weight. The estimate of Mohamed Nor et al. [71], was limited in the amount of dietary exposure data available at this time. This study demonstrates how to account for particle size distributions and perform data alignment when evaluating exposure in humans. Furthermore, Mohamed Nor et al. [71] provided calculations to estimate the accumulation of lipophilic, plastic-related compounds, such as benzo-a-pyrene, di(2-ethylhexyl)phthalate, and 3,3′,4,4′,5-pentachlorobiphenyl, in human adipose tissue. Their model suggests a modest exposure to chemicals leaching from NMPs of approximately 2% of overall chemical intake [71].

More recently, Chen et al. [19] used data characterizing NMPs in the air to estimate exposure from inhalation, and the ingestion NMPs falling out of the air on to food, termed airborne deposition. Chen et al. [19] also performed data alignment and exemplified the ability to unify data from various size ranges to target a relevant size range for the exposure pathway of interest. For instance, their assumed inhalable fraction size range was 1—10 µm, and the available size fraction that could deposit on food before ingestion was 1—5000 µm, and abundance of NMPs was extrapolated for each pathway, respectively. What Chen et al. [19] did not consider was the amount of NMPs originally present in the food items being consumed. They only considered ingestion of the airborne deposition of particles to the food, overlooking potential ingestion to NMPs present in food from processing or packaging that are different than airborne NMPs. Nevertheless, Chen et al. [19] is an important study because it is the first to probabilistically evaluate the exposure of humans to airborne NMP deposited on food being ingested.

The examples covered have considerable diversity in their methods, resulting in different amounts of estimated human exposure to NMPs. For instance, exposure estimates ranged from zero to billions of particles per year, however, it is important to note the dissimilarities in the approaches used amongst the studies. Several common limiting themes are present throughout these examples, such as using a narrow set of exposure media data, a disregard for the quality (i.e., QA/QC) of exposure data, the inclusion of unrealistic assumptions, and no consideration of particle size range differences in the underlying data. Nevertheless, recent studies have advanced the field by implementing behavioural, demographic, and global factors to improve the realism of their estimates [62, 136]. Other examples, such as Mohamed Nor et al. [71] and Chen et al. [19], stand out because of their methodological rigor and implementation of data alignment, probabilistic approaches, and standardizing the particle size distributions, underscoring the need for data harmonization and consistency in exposure assessments. Nevertheless, improvements to these approaches can be made as new data and information becomes available. Adopting these best practices with updated data and insights on important exposure media, pathways, and routes, will significantly enhance the comparability and reliability of future exposure assessments for NMPs.

### Demographic-based exposure scenarios – complexities & prioritization

Here, a perspective and commentary on exposure scenarios for various demographic subgroups of the human population is given, including a prioritization of which exposure scenarios should be investigated. Various elements of the exposure scenarios, possible limitations, and a way forward to surpass the limitations are provided where possible. Notably, the relationships discussed between exposure scenarios, exposure routes, exposure media, and activity factors serve as contextual guidance. Adaptations should be implemented as new evidence or professional opinion suggest it.

**Fig. 2:**
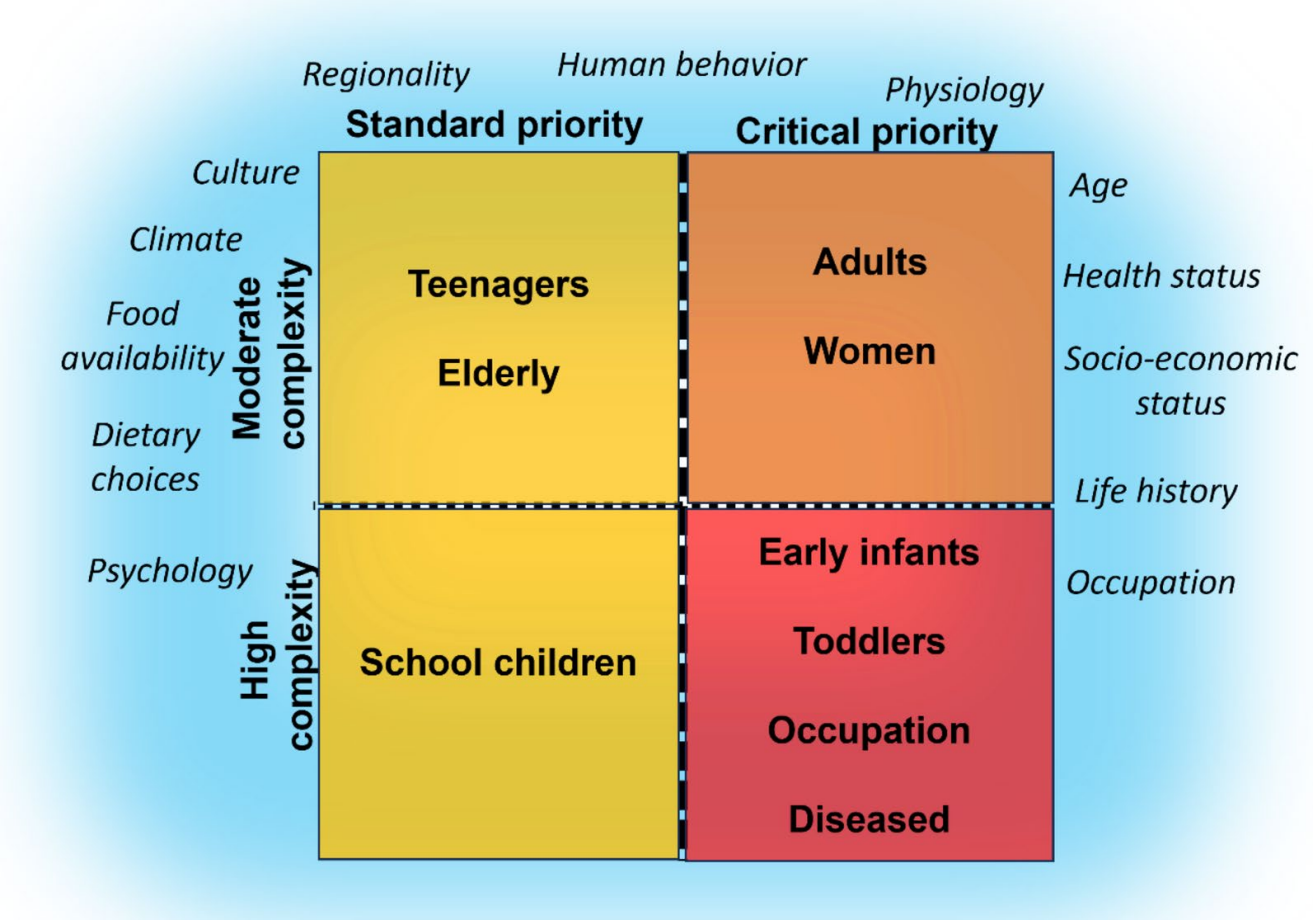
Illustrative two-dimension grid that is separated into four quadrants: top left – Moderate complexity, Standard priority [yellow]; top right – Moderate complexity, Critical priority [orange]; bottom left – High complexity, Standard priority [yellow]; and bottom right – High complexity, Critical priority [red]. Inside each quadrant are the exposure scenarios. For the exposure scenario term “Occupation”, the exposure scenario is for individuals working in hazardous occupations where concentrations of airborne NMPs are relatively elevated compared to typical occupations. For the exposure scenario term “Diseased”, the exposure scenario is for individuals with an underlying disease. The blue cloud represents aspects that have an impact on all exposure scenarios

The exposure scenarios discussed are categorized according to their complexity (moderate or high) and their priority (standard or critical) (Fig. 2). The aim here is to spur a discussion around which demographic groups warrant priority characterization of exposure to NMPs. Complexity is used as a reference to the deficiencies and challenges faced when evaluating exposure because of data limitations/availability, such as under characterized exposure media for relevant pathways, and indefinite activity and physiological factors. A high complexity means that the exposure assessment has a greater number of deficiencies, limiting the reliability of the output. A scenario could be considered as moderate complexity once exposure and activity factors are acceptably defined, meaning there is sufficient data available characterizing NMPs (e.g. number, size, surface area, volume, concentration, etc.) in the relevant exposure media, and the studies providing exposure data are reliable based upon QA/QC protocols. If at least one of the aforementioned aspects is lacking, scenarios become more complex because of inconsistencies in the data and difficulties incorporating variability in the exposure and/or activity factors.

Priority is determined by the urgency needed for evaluating exposure because of anticipated exposure level and concern surrounding health impacts to the exposure demographic. Critical priority exposure scenarios are exposure predictions that are needed as soon as possible to support the human health risk assessment of NMPs. Standard priority exposure scenarios are complementary and likely provide less weight-of-evidence towards establishing regulatory exposure limits or guidelines for exposure to NMPs.

### Proposed exposure scenarios

#### Exposure scenarios for adults

A critical priority is assigned to exposure scenarios for adults because their anticipated exposure provides an estimate applicable to the people globally and can account for a lifetime of exposure. Moreover, there is not yet an exposure assessment that fully incorporates all relevant exposure routes and exposure media, however there are adult scenarios that cover large fragments of the global population according to country [136]. A moderate complexity is given to adult scenarios because the exposure pathways are well known, and there are exposure factor and activity factor data available to integrate in the exposure prediction. A complete description and characterization of NMPs within all relevant exposure pathways and routes should be provided before implementation into the exposure scenario. Without quality data to inform the distribution and characteristics of NMPs that are part of the ingestion or inhalation exposure routes, misleading representations of exposure can occur. The importance of developing and using QA/QC frameworks must be stressed, which consider the reliability and usefulness of the exposure measurements made across different studies.

Exposure assessments for NMPs are often made for adults in a generalized fashion that can predict a typical or central tendency of exposure for adult individuals on a global basis. Evaluating exposure across a general population of adults can serve as a helpful starting point to gather an understanding of the exposure, however it is challenging to conclude that enough data is available for all populations to be covered in such predictions. The uncertainty in the prediction may be overcome using safety factors. Regulatory accepted datasets that describe the activity factors of humans are available from the European Food Safety Authority [31, 32], the European Commission Joint Research Centre’s ExpoFact database [131], and the United States Environmental Protection Agency [104]. This information can be used to define food consumption patterns and rates, physiological information, and activity patterns related to where people spend their time.

General population exposure scenarios for adults provides several advantages. First, the scenario is feasible to implement because it can incorporate meta-analyses and datasets, potentially covering substantial portions of the global population. The variability in activity factors related to geographical location, cultural differences in behaviour, and physiology across age groups is smoothed out. Furthermore, pathway-specific exposure factors and intra-population variability can be captured aggregately, providing a macroscopic view of typical exposure. As a first screening of exposure, insight can be gained about activity factors, exposure factors, or combinations thereof that dictate enhanced or suppressed exposure in the average person.

While useful to an extent, exposure scenarios for adults must be interpreted with caution when they are made in a generalized way, unless exposure is demonstrated to be appreciably lower than adverse effect thresholds. The chronic exposure to NMPs also complicates the selection of activity factors and exposure factors, as individual behaviour impacting their exposure can shift significantly throughout a person’s life, based on age, lifestyle, and where they are living. The information used to define how much time people spend in various indoor or outdoor locations, the types of activities they partake in, and the patterns behind the food and beverages they are consuming, might impact the exposure prediction appreciably.

#### Exposure scenarios for women

Exposure scenarios for women are a critical priority with moderate complexity. Concern about exposure to NMPs leading to adverse health effects in the mother justifies this scenario as a critical priority. It should be noted that women share many commonalities in exposure routes and exposure media with adults (see Table 1) which eases complexities related to defining exposure pathways. However, there is rising concern for pregnant and breastfeeding mothers because accumulation of NMPs could lead to exposure of their fetus or early infant [21, 98]. Furthermore, physiological and behavioural characteristics for women when pregnant may shift, highlighting the need for a tailored exposure scenario to understand how exposure may differ from the general population [76, 128, 138]. Outputs from this scenario could provide insight regarding the activity and/or exposure factors influencing potential increases or decreases in exposure, as well as an identification of possible mitigatory measures to avoid relatively high levels of exposure. For example, in a study on inhalation exposure by Eberhard et al. [30], pregnant women were calculated to inhale more NMPs on a daily average compared to general adults. Eberhard et al. [30] explained that this could be due to the elevated inhalation rate of women during pregnancy, which exemplifies the sensitivity of exposure predictions to small changes in the exposure factors.

Furthermore, physiological changes associated with pregnancy compel elevated caloric intake of up to 10% greater than the average person [50], therefore dietary regimens will shift towards slightly above average food and beverage consumption [57]. Behaviour might also change, such as more focus on the nutritional quality of food and drink being consumed, which might lead to increased vegetable and fruit consumption [39, 102]. Certain pregnancy dietary regimens recommend the consumption of 2 weekly portions of marine fish and an avoidance of raw animal-based foods (e.g., unpasteurized milk) [50]. As well, recommendations include the elimination of alcoholic beverage consumption and a decrease in caffeine containing coffees or teas, which can be offset with other beverage consumption from juices, milk, drinking water, or non-alcoholic beer and wine [39]. Bottled water might be consumed more often by those looking to avoid potential tap water contaminants.

Even with evidence demonstrating the physiological and behavioural changes in pregnant women, one must question how to get the data that is needed to quantify these differences in a way that can be included in a more detailed exposure assessment. Are additional studies needed to characterize exposure in a way that the data is representative for a specific continent, country, or sub-population/demographic within a population? And importantly, are exposure scenarios for women from specific locations or populations needed if their exposure does not differ significantly from the general population person? Is this a certainty without exploring both scenarios extensively? Furthermore, the concept of maternal transfer drives the need for understanding the mother’s exposure profile [21, 98]. Physiologically based toxicokinetic models for women could be used to inform predictions of their internal body burden of NMPs, and the potential for offloading NMPs via maternal transfer. These findings would support risk assessments for women and early infants. Physiologically based toxicokinetic frameworks exist for NMPs biodistribution in humans [117], however there has not yet been a sex-specific or reproductive life stage rendition described.

Additionally, important differences in activity and exposure factors could exist amongst women located in different regions of the world. Resolving these differences in the activity and exposure factors for women of different regions of the globe is a difficult task which may be approached on a case-by-case basis, if the data is available and reliable. In this way, the most relevant activity and exposure factors can inform the overall exposure assessment. These factors can also be investigated regarding how much influence (i.e., sensitivity analysis) they have on the overall exposure prediction and compared with other scenarios.

#### Exposure scenarios for elderly individuals

The elderly demographic (ages 65 and over) has endured the longest period of possible exposure to NMPs, as they have lived through the onset of plastic production and subsequent decades of plastic pollution and evolving regulatory controls. Scenarios for elderly individuals are a standard priority, moderate complexity because human health risk assessment efforts should be focused on evaluating the younger demographics who will be exposed for years and decades to come. However, this suggestion does not mean to completely overlook the elderly, and their health risks related to NMPs, but their exposure routes to NMPs are akin to adults, so in the meantime, an exposure or risk assessment for adults could be extrapolated using safety factors to the elderly. For example, an adult scenario involving a chronic exposure to NMPs for 25 years could serve as a fair proxy of the exposure a person might encounter throughout their elderly years (e.g., from 65–90 years old).

What differentiates elderly from being the same as adult scenarios is their behaviour and physiology. Elderly individuals typically consume less calories, are less active, and have slower metabolic capacity [80]. Elderly individuals may also have varying degrees of comorbidities, such as those impacting the body’s metabolic processes, or those that weaken epithelial barriers and membranes, thereby suppressing the protective nature of barriers related to the gastrointestinal tract, lung epithelium, or blood–brain-barrier [82]. This could make certain organ systems in the body more susceptible to particle absorption and translocation.

As a thought experiment, in a hypothetical situation, if 0.01% of particles translocate across epithelial membranes after exposure to NMPs from ingestion or inhalation, and these particles slowly accumulate in bodily tissues or fluids, then adverse responses to exposure might not be observable until later in life. Today’s elderly population could serve as sentinel indicators for internal body burden of NMPs and potential effects on epithelial membranes, thereby providing retrospective insight on the effects of accumulating NMPs over a human’s lifetime. This type of examination could prove more effective than intensely describing the activity and exposure factors to inform a prediction of daily exposure to NMPs for elderly individuals.

#### Exposure scenarios for individuals working in hazardous occupations

Scenarios for those working occupations where airborne NMPs are present is important because chronic inhalation exposure of particulate matter is acknowledged as being hazardous [37, 66, 110]. Occupational exposure scenarios are considered a critical priority because probabilistic frameworks are needed to predict exposure to NMPs amongst those working in environments where hazardous particles are present and inhalable [91]. In this scenario, the focus should be on exposure from inhalation because exposure from ingestion can be described similarly as for adults, with a consideration for swallowed particles in the workplace that occurs during mucociliary clearance. Defining this scenario is highly complex because work environments in different industries could have different compositions of airborne NMPs which remain to be thoroughly described [30]. Additionally, different protective measures or equipment might be implemented in the workplace to reduce exposure to NMPs and other particulate matter, adding another source of variation within occupational scenarios.

Daily exposure occurs over an extended period throughout one’s occupational career, with certain jobs (e.g., construction) requiring a high degree of physical labor, which could lead to higher inhalation rates in workplaces where airborne NMPs are being generated and are available to be inhaled. Consider the chronic nature of occupational exposure which covers time periods potentially across multiple decades. The selection of which inhalation rate(s) are used in the scenario will indeed impact the inhalation exposure prediction. For example, an “at-rest” air inhalation rate [85], of approximately 360L/hour (6L/minute), is increased nearly threefold to 960L/hour (16L/minute) during normal activity, and nearly threefold again during moderate exercise to 2400L/hour (40L/min). Extrapolating the different inhalation rates for 8 h per day, over period of 45 years, which is typical of a person’s career, could produce extremely different exposure intensities compared to those for adults working in less hazardous occupations. Therefore, the type of work being carried out should be accounted for when selecting the inhalation rate(s) used. Inhalation rates can also be described probabilistically using probability density functions and integrated into exposure models to deliver a more realistic description of inhalation rate fluctuations [5].

Workers exposed chronically to airborne NMPs via inhalation during the production or processing of plastic materials have been found to suffer from various pathologic conditions, ranging from reduced respiratory function to colon, lung, or liver cancer [66, 100, 129]. Whether these effects are due to NMPs or other chemicals that are hazardous remains to be solved [74, 130]. Because the toxicity of NMPs is not well defined, comparing these particulates with dust or other particulate matter, such as particulate matter smaller than 10µm or 2.5 µm is difficult. In addition, for NMPs, the potential effects of particle-associated chemicals needs to be also considered.

Occupational scenarios therefore are best made in a site- or industry-specific manner that consider the unique continuum of particle distributions within respective workplaces or professions. Exposure to NMPs might be mediated by protective measures that are already implemented for exposure to particulate matter smaller than 10 µm or 2.5 µm, such as indoor air filtration measures, or the use of masks that filter particulate matter for these sizes. Countries requiring stricter occupational risk-mitigation practices may experience lower exposure levels, whereas jurisdictions with weaker controls could face higher potential exposure. Ensuring the nuances of workplace exposure and safety measures are brought into the exposure scenario adds complexity and is necessary for a relevant exposure assessment. Employing a probabilistic inhalation exposure model presents a valuable opportunity to assess the risks of airborne NMPs, as well as other particulates that are not NMPs, in situations where a relatively higher workplace exposure to particulates is expected [42, 91].

#### Exposure scenarios for individuals with disease

Scenarios for individuals with disease are a critical priority with high complexity and presents an opportunity to learn about the presence or absence of NMPs alongside morbidities. Individuals suffering from diseases related to immune-mediated inflammation, which may present themselves as acute exacerbations or chronic illnesses, as well as other serious, long-term illnesses (e.g., cancer) could limit one’s ability to tolerate exposure to NMPs [106]. Studying the impacts of exposure to NMPs in individuals with disease, which could span from early infants to elderly people, provides insights regarding the association of NMPs and disease pathogenesis or etiology. Nano- and microplastic particles have been measured in unhealthy human tissue such as blood clots [115], tumors [17], as well as vital organs [60]. Take the liver for example – a single study [40] exists to our knowledge that has detected NMPs in liver tissue. Samples were taken from patients with some form of liver disease or cirrhosis, as well as healthy patients, and a greater prevalence of NMPs in samples from patients with diseased livers was reported. However, presence does not mean causality, and further investigation here is needed. It should be noted that Horvatits et al. [40] implemented sufficient measures related to QA/QC (e.g., blank correction, reporting limits of detection, and limits of quantification). Their study is therefore a sound example of characterizing size distributions of NMPs within a biologically reasonable size range of 4 to 30µm in tissues derived from healthy and diseased patients.

Several factors distinguish this scenario as being highly complex, even though general exposure routes and media for adults can be transposed in this scenario. As mentioned in the elderly scenario discussion, disease can impact the integrity of epithelial barriers and membranes. If NMPs were to act as vector for a bacterial or viral disease, or chemical exposure, then individuals who have a pre-existing disease would presumably be more sensitive to effects of xenobiotic exposure due to the altered metabolic, biochemical, and genetic state [63]. Conversely, those with hypersensitive immune systems could overreact from exposure to NMPs and lead to unnecessary acute inflammatory responses, similar to an allergic reaction. An example of hypersensitivity to polymers can be found in Beige et al. [10], who explains how approximately 2% of hemodialysis patients show hypersensitivity to polysulfonate. Exaggerated immune responses can also promote an increased permeability of internal membranes within the human body, possibly due to long-term chronic inflammatory responses [69]. Investigating specific adverse health responses, such as liver cirrhosis [40, 106] or inflammatory bowel disease [126], and the association of the disease with the presence of NMPs internally, could be an astute approach for studying the impacts of NMPs in diseased people.

#### Exposure scenarios for early infants (0–1 year old)

Exposure scenarios for early infants are a critical priority because this is arguably the most concerning time-period of one’s life [16, 21]. Other perspectives such as Christopher et al. [21] and Sripada et al. [98] also compel the need for human health risk assessment specific to early infants. High complexity is assigned to this scenario because the exposure routes and exposure media are unique to this age category. For example, exposure from maternal transfer, infant formula, polymer baby bottles, rubber soothers, and inhalation need to be considered together. As well, the endocrine and neural systems, which interact strongly with the immune system, are not fully developed [67]. The increased metabolic, cardiovascular, and breathing rates accompanying neonatal growth [72, 114], and a larger surface area-to-volume ratio, could reasonably alter exposure and absorption rates, and potentially the systemic distribution of NMPs within the body, compared to adults or older children. During early infancy, frequent mouthing of hands and plastic objects can constitute a relevant exposure pathway to NMPs – one that is negligible in older children and adults [98].

For early infants, activity factors should consider the fact that approximately 14–17 h of each day are dedicated to sleeping [103]. For the hours spent awake, it is plausible to reason that the amount of time spent outdoors and indoors is likely to vary country-to-country with some dependence on the region, climate, and/or season. Studies should aim to characterize indoor residential sleeping rooms for early infants. Quantitative frameworks exist for describing inhalation exposure to NMPs in children, including early infants [30]. Inhalation exposure calculations, based on the number of NMPs per kilogram of bodyweight per day, indicate that children globally endure over threefold higher exposure levels than adults [30]. These findings raise concern because inhalation exposure to extrinsic air pollution has been linked to pathologic conditions in the form of adverse pulmonary function (e.g., asthma) and should not be overlooked [35, 83].

Activity factors for exposure through ingestion are not as straight forward because these must consider parental behaviour, as parents will provide the required sustenance to their child. Breastfeeding and infant formula are the major sources of caloric intake for early infants, however mothers in different regions of the world breastfeed at different rates [120]. Countries like Canada, France, and Netherlands have breastfeeding rates of around 10%, whereas other countries like the United States, China, Japan, and India have rates of approximately 35% to 90%, demonstrating a wide variation of Global breastfeeding tendencies [111]. With these factors in mind, dietary exposure pathways for nursing babies can be clearly defined and manipulated in scenarios.

For the ingestion exposure pathway, one can alter the ratio of breastmilk-to-infant formula being consumed for early infants to increase the representativeness of actual consumption tendencies for a given region or country. This has been demonstrated by Büchner et al. [14] and van Rossum et al. [108], who considered different dietary regimens for nursing babies during the first six months, such as being strictly breastfed or fed infant formula, to illustrate the risks of exposure to microbial pathogens and chemicals from these media. By applying this concept for NMPs with aligned data, one could better estimate consumption patterns and proceed towards answering how much exposure to NMPs early infants encounter via ingestion of breastmilk or infant formula. Furthermore, infant formula products exist to prevent “spitting-up” post-feed, to calm upset stomachs, and even those which ask the parents to combine breastmilk and infant formula together to form the final product for consumption. It is important that these niche products are characterized for contaminations with NMPs, alongside typical infant formula, to ensure the safety of these products that provide the nutritional foundation for early infants [44]. Lastly, the flux of NMPs from plastic products, such as rubber baby soothers, baby bottles, toys, and other plastic items commonly used by early infants should be investigated and brought into the exposure scenario.

#### Exposure scenarios for toddlers (ages 1–4)

Exposure scenarios for toddlers are regarded as a critical priority scenario with high complexity, even though the exposure pathways and media can be described similarly as school children. The high complexity of this scenario stems from the rapid physiological growth and developmental aspects of toddlers, as well as their unique behaviour. Toddlers might also be exposed to NMPs from dust and soil that typically older children, teenagers, and adults would not encounter [98]. As well, toddlers might also practice mouthing of objects similarly to early infants [98]. The impact of activity factors to exposure through these media are difficult to describe because children will crawl, play, and unintentionally inhale or ingest soil and/or dust differently [98]. Further difficulties arise when selecting the appropriate exposure and activity factors to describe toddlers, as parenting patterns will also influence where and how toddlers spend their time, as well as the types of foods they are provided with. Additionally, there could also be a regionality or cultural influence on all of this. Scenarios for toddlers should implement as much toddler specific activity and exposure factor information as possible to inform the exposure prediction.

#### Exposure scenarios for school children (ages 5–13)

Exposure scenarios for school children are assigned a standard priority because there is a need for a comprehensive exposure assessment for this age group. This scenario is considered as highly complex even though the exposure routes and media are defined and comparable to teenagers, and behavioural information for this age group is available for use in exposure assessments [6, 73]. The added complexity comes from the extensive physiological growth and development compared to during adulthood, which could relatively increase their exposure to NMPs in food. The distinctive exposure routes and media for school children also add complexity to the scenario, as children will frequent areas like schools, playgrounds, and daycare centers where adults (other than those teaching or supervising the children) would typically spend less time. It is also difficult to capture the variability in parenting psychology and how this impacts a school child’s exposure to NMPs, as this could shift the dietary regimens and types of activities they are involved in.

#### Exposure scenarios for teenagers (ages 13–18)

Teenagers are a demographic that requires targeted exposure assessment because of their changing behavioural patterns. This scenario is considered as a standard priority and moderate complexity. Similarities to adults exist across exposure pathways and dietary consumption patterns. In terms of physiology, teenagers go through substantial developmental processes (e.g., puberty) and growth during their teenage years. The underlying immune, neuronal and metabolic systems of teenagers are more developed than school children, and this could have implications on sensitivity to inhaled or ingested NMPs [84]. Therefore, exposure scenarios developed for adults may also adhere to teenagers given the implementation of safety factors and the variability in the underlying data. Teenagers also differ from school children because they live more autonomously, making their behaviour less predictable. Teenagers might engage in more diverse social environments; therefore, the development of teenage-specific activity factor data is needed for an accurate exposure estimation.

### Advancing exposure assessment practices for NMPs

Advancing exposure assessment practices requires a multifaceted approach that addresses key gaps in current approaches and methodologies. This section outlines three critical strategies to enhance the robustness and relevance of exposure assessments: thoroughly characterizing exposure pathways in exposure scenarios; aligning multidimensional exposure data to ensure coherence across exposure sources; and, applying probabilistic approaches to better account for variability and uncertainty in exposure estimates while linking external-internal exposure. In unison, these strategies contribute to more transparent, reliable, and science-driven risk assessments.

#### Thorough characterization of external exposure

Providing a comprehensive description of external exposure provides better information for making estimates of internal exposure. This requires a thorough characterization of NMPs in the relevant exposure media composing each of the oral (ingestion) and inhalation pathways. Additionally, exposure to NMPs during intravenous administration of medicine requires further investigation to understand the fluxes of this exposure route [59].

Importantly, external exposure from the consumption of food and beverages and the presence of NMPs across various food matrices has been well documented [112]. However, this pathway encompasses several distinct aspects that warrant careful delineation because NMPs are not always found inside of food items. The fallout of airborne NMPs onto food [19, 77] represents an often-underappreciated process through which contamination may occur during food preparation and consumption. In Chen et al. [19], the airborne deposition of NMPs onto food that is consumed was estimated to cause oral exposure, based on particle count, comparable to that of inhalation of airborne particles. Notably, when exposure was assessed based on mass, rather than particle count, exposure was found to be approximately three orders of magnitude higher than that from inhalation, owing to the ingestion of larger particles that are unlikely to be inhaled [19]. The importance of incorporating the airborne deposition of NMPs onto food and beverages into assessments of the oral exposure pathway must be emphasized. As well, the larger particles inhaled, trapped, and swallowed during mucociliary clearance should be considered.

Another process that can lead to oral exposure of NMPs is the use of plastic packaging, containers (e.g., tupperware), and films or food wraps (e.g., saran wrap, low density polyethylene films). During use, plastic packaging and films often endure harsh processes such as freezing, repeated freeze–thaw cycles, microwave irradiation, and high temperatures, which promotes fragmentation and generation of NMPs [116], resulting in the contamination of food and beverages. Items found in the kitchen, such as those used in food preparation (e.g., cutting boards), and equipment used for cooking, serving (e.g., plastic ladles, spatulas and utensils), and cleaning (e.g., microfiber clothes, sponges), can potentially shed NMPs and contaminate food and cause oral exposure [65, 97].

Plastics also cause exposure in early infants and toddlers who often interact closely with plastic baby bottles and soothers, including the mouthing of plastic toys or objects [99]. Plastic products have been shown to release NMPs during the disinfection process, leading to surface-active particles which can cause direct exposure when placed in a babies mouth [99]. Toddlers may also have an elevated consumption of soil and dust from crawling, playing outdoors, and behaving as children do [79]. Oral exposure to NMPs from these media should not be overlooked when evaluating the overall external exposure of children in these age groups.

Furthermore, the consumption of infant formula (e.g., baby formula, formula milk) by early infants, as well as breastmilk, are important exposure media to consider, which are less relevant to older children, except for perhaps toddlers consuming toddler formula. For infant formula, contamination with NMPs may occur during the manufacturing process [44]. Kadac-Czapska et al. [44] directly measured an average of approximately 420 NMPs per 1000 g of infant formula to assess contamination of the product itself, excluding any preparatory factors. It has been hypothesized that during formula preparation by the parents, which involves hot temperatures (i.e., boiling water) and shaking, abrasion of polypropylene enters the infant formula. After preparation, Li et al. [58] found approximately 4,000,000 NMPs per liter of prepared infant formula. Note, the intention here is not to compare the findings of these studies because the data is not aligned, and this is not the goal of this current evaluation. Rather, it should be highlighted that more studies evaluating how NMPs contaminate infant formula and breastmilk, and the degree of contamination are needed. For example, studies have also measured NMPs in breastmilk, however the underlying mechanisms of particle translocation into the breastmilk remain unclear. And no evidence is available on the potential flux of NMPs contaminating breastmilk after the use of plastic-based breast pumps.

Nano- and microplastic particles are routinely detected in outdoor air, both as suspended particles and as airborne deposition, across diverse environments globally [19, 78]. These include urban, rural, industrial, and remote locations, including mountainous regions and oceanic areas [12, 15, 45, 81, 118]. Furthermore, NMPs are also similarly detected in indoor air, across numerous settings such as households, schools, and various workplaces (e.g., offices, hospitals, laboratories) [61, 125].

The importance of distinguishing between human exposure to NMPs originating from outdoor versus indoor air has been emphasized by Eberhard et al., [30]. Indoor air has been demonstrated to contain higher concentration of NMPs compared to outdoor air, resulting in increased human exposure [30, 68]. Evidence from China suggests indoor concentrations being up to approximately eight-fold higher than outdoors for their study locations [61]. Furthermore, airborne deposition indoors occurs more substantially than outdoors [19, 135]. The potentially elevated risk associated with contaminated indoor air has been highlighted by the European Commission’s Joint Research Centre particularly given that individuals typically spend upwards of 90% of their time indoors [53]. It should be noted that the Kotzias [53] study focused primarily on benzene and not NMPs, however, NMPs should indeed be considered alongside other priority substances present in indoor air.

Airborne exposure to NMPs in occupational settings is also important to be mindful of. The demographic of individuals working in occupational settings where airborne particles with greater hazardous potential are present could be exposed at different intensities depending on the type of work being performed. Landfills, construction sites, metal smelting, plastic production and processing facilities, nanomaterial manufacturing, and textile factories are all recognized as hazardous locations where workers are exposed to elevated air pollution in the form of particulate matter smaller than 10 µm or 2.5 µm, yet the characterization of NMPs contribution to the particulate matter air pollution in these workplaces globally is lacking [2, 64, 75, 95]. Occupational exposure can also occur in less recognized environments, such as for dental specialists who may inhale a variety of inorganic and plastic-related particulates during their work activities [36]. Further studies are needed that evaluate the presence and types of NMPs in different indoor air settings (e.g., bedroom, kitchen, living room, school, hospital, indoor factories, etc.) to help provide a better understanding of exposure indoors.

#### Multidimensional exposure data alignment

A challenging factor when comparing the exposure estimations from different studies is that they all use different combinations of exposure data. With each set of underlying exposure data being generated through varying methodologies – from study design to sample quantity, to the analytical method used, there is no harmonization of the particle size ranges or the particle properties evaluated in the different studies. Foremost, if an exposure assessment uses data from different exposure studies and does not consider the differences in the particle size ranges measured between the studies, inaccuracies in the exposure estimate are possible [47]. These inaccuracies arise because studies applying quantification methods with a lower size detection limit will count more particles, since the size distribution of NMPs is well described by a power law relationship [47]. Studies also report different dose metrics, such as particle counts (or number) per volume of air or water, or per mass of food, as well as concentration data (e.g., µg/g or mL). Converting from a particle number concentration to volume and/or mass concentration (or vice versa) should consider the wide range of sizes, shapes, and densities of NMPs, and not rely on assumptions that particles humans are exposed to are simply spheres. Several steps can be taken to harmonize the different data sets used in exposure assessments and scenarios. Calculation methods to align and unify data, facilitating more appropriate comparisons of studies using different particle characterization methods, and reporting different particle metrics, have been published previously [9, 47, 51, 52]. By using these alignment approaches, unified data can be used in the exposure assessments (Table 2).

**Table 2:** Aligning data targeting different size ranges: how to determine CF, correction factor

$$CF = \frac{{\int_{{x_{1D} }}^{{x_{2D} }} b x^{ - a} }}{{\int_{{x_{1M} }}^{{x_{2M} }} b x^{ - a} }} = \frac{{x_{2D}^{1 - a} - x_{1D}^{1 - a} }}{{x_{2M}^{1 - a} - x_{1M}^{1 - a} }}$$
Variable “x” denotes size and variable “*b*” denotes a fitting parameter (Kooi and Koelmans, 2019). α = power law slope. Values for α should be estimated from measurements for respective exposure pathways (Mohamed Nor et al., 2021). Subscripts 1 and 2 are the minimum and maximum values of the range (µm). Variables “D” and “M” are the default and measured ranges, respectively. CF is the correction factor which can be used to translate measured size ranges to a size range of interest, such as the full size range of NMPs, or a size range deemed biologically plausible, as needed or appropriate (Koelmans et al., 2020).
Example calculation: To re-scale a range from 30 to 2000 µm to the range of 1 to 5000 µm, the values for x_1D_, X_2D,_X_1M_, and X_2M_, are 1, 5000, 30, and 2000, respectively.
α (power law slope) = 1.6. If the measured number concentration for the range from 30 to 2000μm is 100 particles/L and CF = 8.32, the extrapolated number concentration for the range from 1 to 5000 μm would be 8.32 × 100 = 832 particles/L (example calculation from Koelmans et al., 2020).

Examples of data alignment and rescaling exist in the literature for environmental risk assessment, such as in Redondo-Hasselerharm et al., [90] and have yet to be applied in a comprehensive human health risk assessment. An important feature of these studies is that the toxicologically relevant metric of NMPs can be described using a probabilistic density function. This provides a continuous distribution of particles for that characteristic, allowing the collective exposure contribution of those particles to be probabilistically linked to the toxicological implications for the same characteristic.

Consider that the mechanisms of toxicity for endpoints such as cytotoxicity, or inflammatory responses, are potentially driven by different toxicologically relevant metrics of NMPs, like size, surface area, specific surface area, volume, shape, or aspect ratio. Exposure assessments should evaluate multiple metrics where practicable. The distribution of toxicologically relevant particle characteristics in different exposure media can be described within a respective continuum for that characteristic. For example, a hypothetical size continuum for NMPs could be defined as 0.01—5000 µm; or, for surface area, as 0.003–30 mm^2^; or additionally, taking mass into account, for specific surface area, as 0.0012–60 m^2^/g. If the data used to compose the continuum was from studies measuring different size ranges, or other metrics, the data should be aligned, based on power law distributions from data for the respective exposure media. Doing so enables probabilistic estimates and the translation of a collection of exposure data into more comparable dose metrics – allowing translation to an internal exposure dose and further comparisons to hazard data for the same particle characteristic. Koelmans et al. [48] provides a comprehensive discussion and example of this concept.

A reliable exposure assessment is available in the aforementioned Mohamed Nor et al. [71] study, which rescaled different exposure data and used probabilistic approaches to quantify and communicate the uncertainty. In Mohamed Nor et al. [71], the exposure data was not compared to any hazard data, therefore no risk characterization was made. Today, frameworks such as Koelmans et al. [48] exist for connecting exposure-to-effect data, using the continuum approach anchored in probability density functions. Additionally, there are studies [70, 136] providing new data on the presence of NMPs in various components of the human diet missed in Mohamed Nor et al., [71]. And, complementary data characterizing NMPs in outdoor and indoor air environments [30], and insights on which toxicologically relevant metric(s) are important [49, 94] are available. The human exposure approaches used by Mohamed Nor et al. [71] should be revisited, adapted, and applied to the new data available today, providing a more robust, updated estimate of human exposure to NMPs. One adaptation would be to include the exposure contribution of airborne deposition of particles to food in similar fashion to Chen et al. [19].

#### Probabilistic approaches to link external and internal microplastic exposure

Each day, humans are exposed to the polydisperse nature of NMPs which vary in size, shape, surface area, mass, and density. Ultimately, the total external exposure to NMPs serves as an input parameter for physiologically based toxicokinetic models to estimate an internal biodistribution of NMPs across different organs and bodily fluids, providing a quantification of total body burden and internal exposure. A distinction between external and internal exposure to NMPs has been framed in a quantitative risk assessment framework by Wardani et al. [117]. The wide range of external exposure possibilities highlights the complexity of quantifying the total intake of NMPs for humans. This heterogeneity, combined with inconsistencies in definitions and analytical techniques, introduces substantial variability in reported concentrations across studies [89].

To address this challenge, Monte Carlo simulations and probabilistic methods are increasingly favored [19, 90]. Mohamed Nor et al. [71] first proposed the use of probability density functions to represent the particle size distributions within each intake medium for human health exposure assessment. For example, Mohamed Nor et al. [71] demonstrated that NMPs concentrations in air and food by particle size follow power law distributions, where smaller particles are more abundant. Additionally, data harmonization techniques such as size realignment, among other characteristics of NMPs are essential for unifying heterogeneous datasets into a consistent, size-resolved exposure profile [47, 48]. These characteristics of external exposure distribution and size realignment using the probability density functions approach also should be considered when estimating the internal concentration of NMPs in humans [117].

Although humans are exposed to a broad range of particle sizes, only a fraction of NMPs are capable of translocating across biological barriers. Recent findings by Chen and Lin [20] identified at least seven physiological barriers relevant to NMPs transport in the human body: the intestinal, pulmonary, blood–brain, placental, blood-testis, blood–breastmilk, and skin barriers. Transport across these barriers is also governed by size-dependent mechanisms such as intracellular trafficking (e.g., phagocytosis, micropinocytosis) and paracellular transport [88, 123]. This barrier selectivity significantly influences internal exposure distribution.

The use of probabilistic approaches and physiologically based toxicokinetic models to translate external exposure data into internal concentrations provides a more mechanistic and realistic understanding of NMPs behaviour in the human body. It captures variability in exposure sources, particle characteristics, and interindividual diversity. Given the particle-specific nature of NMPs, exposure assessment and risk characterization must move beyond simple mass-based metrics, adopting probabilistic methods to accurately assess potential health impacts.

### Summary & forward thinking

Exposure scenarios for NMPs serve as flexible frameworks to describe exposure for specific demographics within human health risk assessments. Regardless of how general or targeted the exposure scenarios are, they should reflect demographic-specific age, behavioural, and physiological factors, as well as regional and/or cultural aspects, to reliably inform the exposure predictions. Additionally, exposure scenarios should aim to include often overlooked contributors to exposure – including air-to-food deposition, plastic packaging and films (including interactions with plastic products in general), and in the case of early infants, maternal transfer.

Data informing exposure scenarios also contain a series of underlying uncertainties associated with the activity and exposure factor information that are used. These can be assumptions related to dietary patterns and/or inhalation rates for individuals. As well, there is variability and uncertainty throughout exposure data for NMPs in the media causing exposure, stemming from inconsistencies in study design and methods related to the quantity (e.g. mass or volume) of samples measured, replication, analytical instrumentation, and targeted particle size ranges. Other QA/QC aspects should also be considered, such as the use of blanks, positive and negative controls, and appropriately correcting for blank contamination as appropriate. Addressing these challenges requires rigorous QA/QC practices, standardized reporting criteria, and consistent methodologies for multidimensional data alignment (see section on “ Multidimensional exposure data alignment”). Doing so allows for studies to be interpreted and compared with greater consistency and reliability.

Focused attention on insufficiently characterized yet potentially significant exposure media —such as staple dietary items (e.g., meat, dairy products, oils and fats, cereals, grains, fruits, and vegetables) and airborne particles within residential other indoor settings (e.g., schools, hospitals, potentially hazardous occupational jobs) — is particularly necessary [19, 30, 101]. For instance, NMPs have not been thoroughly characterized in food items that compose meat, dairy products, fruits, vegetables, cereals and grain products, which even if consumed less frequently could contribute to human exposure if their concentration of NMPs is relatively high. Additionally, the characterization of airborne NMPs across various indoor residential locations (e.g., kitchen, bedroom, dining room, etc.), where people spend most of their time and exposure concentrations are greater compared to the outdoors, are urgently needed to make better estimations of exposure [19, 30]. For children, especially early infants and toddlers, their interaction with plastic products (e.g., rubber soothers, baby bottles, toys) should be considered in the context of oral exposure.

To transparently manage uncertainties and variability in activity and exposure factor data, adopting probabilistic approaches is strongly recommended. Probabilistic methods, incorporating multidimensional data alignment and percentile-based (e.g., median, 95th percentile) exposure data, enable more precise estimations of exposure distributions and facilitate sensitivity analyses, ultimately enhancing the reliability of human health risk assessments [23, 32]. Using a probabilistic approach can allow for specific parts of the exposure factors and activity factors to be used by risk assessors. For example, a worst-case assessment could be performed using exposure and activity factor data from the 95th percentile [23]. Or, a tiered approach, such as using the median of exposure data could be done [32]. In this way, the data informing the exposure scenarios can be handled probabilistically in the quantitative assessment to provide transparency behind the uncertainty of the data. Moreover, probabilistic approaches can bridge external exposure predictions with internal body burden assessments, accounting for physiological mechanisms and particle-specific toxicokinetic processes [117]. Furthermore, there could be potential to estimate one’s personal or individualized external exposure, using data curated specifically around a person’s activity and exposure factors. Inputting this data to a physiologically based toxicokinetic model, that is perhaps also “personalized” for that individual, based on age and physiology, information on where NMPs are translocating and distributing within the body can be gathered. The internal body burden concentrations could be evaluated to adverse effect thresholds for different organs, and potentially connected to an appropriate adverse outcome pathway.

Moving forward, exposure scenarios must evolve continuously as new data emerges, emphasizing demographic-specific scenarios that reflect real-world complexities [19, 21, 136]. Capturing the multitude of differences amongst demographics is difficult yet integral for improving exposure accuracy and reducing generalized assumptions that limit risk assessment validity. Confronting the challenges associated with assessing and linking external-internal exposure to NMPs supports the ultimate goal of human health risk assessment – to protect public health.

To conclude, the following suggestions are provided:

- Exposure scenarios across different demographics should be strategically prioritized based on assessment complexity and degree of concern associated with each group.
- Exposure scenarios should define the relevant activity factors related to the physiological parameters (e.g., body weight, age, gender), food consumption rates/patterns, inhalation rates, and behaviour of the exposure demographic.
- Exposure scenarios should define the relevant exposure factors related to exposure duration, exposure frequency, and the characterization of NMPs (e.g., concentration and properties) in the appropriate exposure media for the demographic of interest.
- Exposure from the inhalation of air and the ingestion of food and beverages should be comprehensively described, including contamination from air-to-food deposition, as well as from food packaging and films, when applicable, and if the available data makes it possible.
- Exposure scenarios for early infants should consider exposure via maternal transfer.
- The QA/QC of exposure data should be evaluated to gain an understanding of how reliable and useful the data is for assessing exposure.
- Exposure data from different sources should undergo multidimensional alignment to enable transparent, credible comparisons of data across studies, and linkages of toxicologically relevant metrics for NMPs across exposure and effects data.
- Probabilistic approaches incorporating aligned data should be used to describe external exposure and inform physiologically based toxicokinetic models, enabling estimates of internal body burden (i.e., internal exposure) and a connection between exposure and effects data for toxicologically relevant metrics.

## Supplementary Information

Supplementary Material 1.

## Data Availability

No datasets were generated or analysed during the current study.

## Abbreviations

CF: Correction factor
kg: Kilogram
g: Gram
mL: Milliliter
mm: Millimeter
nm: Nanometer
NMPs: Nano- and microplastic particles
QA/QC: Quality assurance/quality control
U.S. EPA: United States Environmental Protection Agency
WHO: World Health Organization
µm: Micrometer
