# Supplementary material for: Exposure scenarios for human health risk assessment of nano- and microplastic particles
Source: Microplast nanoplast. 2025 Jul 14;5(1):28. doi: 10.1186/s43591-025-00134-9 (PMC12259766; doi:10.1186/s43591-025-00134-9)
Supplement: Supplementary file 1 — Supplementary Material 1. [file 43591_2025_134_MOESM1_ESM.docx]

**Supplementary information**

**Exposure scenarios for human health risk assessment of nano- and microplastic particles**

Taylor Lane^1*^, Ira Wardani^1^, Albert A. Koelmans^1^

*^1^*Aquatic Ecology and Water Quality Management Group, Department of Environmental Sciences, Wageningen University, 6700 AA Wageningen, The Netherlands

* *Corresponding* author: [taylor.lane@wur.nl](mailto:taylor.lane@wur.nl)

Table S1: Summary of exposure assessment and scenario studies that provided estimations of human exposure to NMPs.

| Reference | Exposure demographic | Exposure routes | Exposure media | Data alignment? | Probabilistic approach? | Exposure prediction |
| --- | --- | --- | --- | --- | --- | --- |
| Cox et al., (2019) | Adults (≥19 years old)  Children (1-18 years old) | Ingestion & inhalation | Seafood, sugar, honey, salt, alcohol, bottled water, tap water, air | No | No | 74,000 - 114,000 particles/year |
| Conti et al., (2020) | Adults  Children | Ingestion | Fruits & vegetables | No | No | 460,000 - 1,400,000 particles/kg bodyweight/day |
| Zhang et al., (2020) | Adults | Ingestion & inhalation | Salt, seafood, drinking water, dust, indoor air, outdoor air, | No | No | 0 - 30,077,700 particles/year |
| Domenech et al., (2021) | Adults | Ingestion, inhalation & dermal | Fruits & vegetables, seafood, bottled water, salt, alcohol, air | No | No | 29,300,000,000 particles/year |
| Senenthirajah et al., (2021) | Adults | Ingestion | Shellfish, fish, salt, honey, sugar, beer, tap water, bottled water, drinking water | No | No | 11,845 - 193,200 particles/year |
| Mohamed Nor et al., (2021) | Adults  Children | Ingestion, inhalation | Fish, mollusc, crustacean, tap water, bottled water, salt, beer, milk, air | Yes | Yes | Adults: 31,500 - 61,320,000 particles/year  Children: 19,000 - 50,000,000 particles/year |
| Bai et al., (2022a) | Adults | Ingestion | Seafood, poultry, honey, salt, sugar, beer, wine, bottled water, milk, other beverages | No | No | 142,000 - 154,000 particles/year |
| Bai et al., (2022b) | Toddlers (2-5 years old)  Children & Teenagers (6-17 years old)  Adults (18-59 years old)  Elderly (>60 years old) | Ingestion | Vegetables, rice, noodles, tofu, meat, bubble tea, coffee | No | No | Toddlers: 8,840 - 17,680 particles/year  Children & Teenagers: 6,708 - 3,416 particles/year  Adults: 8,008 - 15,964 particles/year  Elderly: 6,760 - 13,520 particles/year |
| Zuri et al., (2023) | Adults, infants & newborns | Ingestion & inhalation | Milk, fish, wine, shrimp, mussels, beer, indoor air, outdoor air | No | No | Adults: 13.6 - 417 (particles/kg bodyweight/day)  Infants: 0.07 - 338 (particles/kg bodyweight/day)  Newborn: 0.14 - 1120 (particles/kg bodyweight/day) |
| Chen et al., (2024) | Adults,  Children | Ingestion  &  inhalation | Airborne deposition to food, outdoor air, indoor air | Yes | Yes | Adults: 548,600 particles/year  Children: 483,600 particles/year |
| Eberhard et al., (2024) | Adults (≥21 years old), pregnant women (second trimester), adolescents (11- <16 years old), young children (2- <6 years old), infants (birth to <1 year old). | Inhalation | Indoor air [passive] – residential, school, workplace | No | No | Adults: 1,432,990 particles/kg bodyweight/year  Pregnant women: 2,192,190 particles/kg bodyweight/year  Adolescents: 2,704,285 particles/kg bodyweight/year  Young children: 4,357,370 particles/kg bodyweight/year  Infants: 5,354,915 particles/kg bodyweight/year |
| Liu et al., (2024) | 1-4 years old  5-11 years old  12-17 years old  18-64 years old  >64 years old | Inhalation & ingestion | Indoor air, outdoor air, dust & soil | No | No | 1-4 years: 57,361,500 particles/year  5-11 years: 5,258,500 particles/year  12-17 years: 42,698,000 particles/year  18-64 years:60,503,000 particles/year  >65 years: 43,577,000 particles/year |
| Milne et al., (2024) | Adults | Ingestion | Fish, chicken, meat, shrimp, pork, plant-based meat, tofu, | No | No | 1500 particles/year  (protein consumption only) |
| Zhao and You (2024) | Country | Ingestion, inhalation | Food (all categories), outdoor air, indoor air | No | No | Inhalation: 3,650,000 - 1,040,615,000 particles/year (inhalation per person, country level)  Dietary intake: 14,700 - 156,600 mg/day (ingestion per person, country level) |

References:

Bai CL, Liu LY, Hu YB, Zeng EY, Guo Y. Microplastics: A review of analytical methods, occurrence and characteristics in food, and potential toxicities to biota. Science of The Total Environment. 2022a Feb 1;806:150263.

Bai CL, Liu LY, Guo JL, Zeng LX, Guo Y. Microplastics in take-out food: Are we over taking it?. Environmental Research. 2022b Dec 1;215:114390.

Chen Y, Meng Y, Liu G, Huang X, Chai G. Probabilistic Estimation of Airborne Micro-and Nanoplastic Intake in Humans. Environmental Science & Technology. 2024 May 15.

Conti GO, Ferrante M, Banni M, Favara C, Nicolosi I, Cristaldi A, Fiore M, Zuccarello P. Micro-and nano-plastics in edible fruit and vegetables. The first diet risks assessment for the general population. Environmental Research. 2020 Aug 1;187:109677.

Cox KD, Covernton GA, Davies HL, Dower JF, Juanes F, Dudas SE. Human consumption of microplastics. Environmental science & technology. 2019 Jun 5;53(12):7068-74.

Domenech J, Marcos R. Pathways of human exposure to microplastics, and estimation of the total burden. Current Opinion in Food Science. 2021 Jun 1;39:144-51.

Eberhard T, Casillas G, Zarus GM, Barr DB. Systematic review of microplastics and nanoplastics in indoor and outdoor air: identifying a framework and data needs for quantifying human inhalation exposures. Journal of exposure science & environmental epidemiology. 2024 Mar;34(2):185-96.

Liu K, Li Q, Andrady AL, Wang X, He Y, Li D. Underestimated activity-based microplastic intake under scenario-specific exposures. Environmental Science and Ecotechnology. 2024 Mar 1;18:100316.

Milne MH, De Frond H, Rochman CM, Mallos NJ, Leonard GH, Baechler BR. Exposure of US adults to microplastics from commonly-consumed proteins. Environmental Pollution. 2024 Feb 15;343:123233.

Mohamed Nor NH, Kooi M, Diepens NJ, Koelmans AA. Lifetime accumulation of microplastic in children and adults. Environmental science & technology. 2021 Mar 16;55(8):5084-96.

Senathirajah K, Attwood S, Bhagwat G, Carbery M, Wilson S, Palanisami T. Estimation of the mass of microplastics ingested–A pivotal first step towards human health risk assessment. Journal of Hazardous Materials. 2021 Feb 15;404:124004.

Zhang Q, Xu EG, Li J, Chen Q, Ma L, Zeng EY, Shi H. A review of microplastics in table salt, drinking water, and air: direct human exposure. Environmental science & technology. 2020 Mar 2;54(7):3740-51.

Zhao X, You F. Microplastic Human Dietary Uptake from 1990 to 2018 Grew across 109 Major Developing and Industrialized Countries but Can Be Halved by Plastic Debris Removal. Environmental Science & Technology. 2024 Apr 24;58(20):8709-23.

Zuri G, Karanasiou A, Lacorte S. Microplastics: Human exposure assessment through air, water, and food. Environment International. 2023 Sep 1;179:108150.
